# Population Pharmacokinetics of Levetiracetam and Dosing Evaluation in Critically Ill Patients with Normal or Augmented Renal Function

**DOI:** 10.3390/pharmaceutics13101690

**Published:** 2021-10-15

**Authors:** Idoia Bilbao-Meseguer, Helena Barrasa, Eduardo Asín-Prieto, Ana Alarcia-Lacalle, Alicia Rodríguez-Gascón, Javier Maynar, José Ángel Sánchez-Izquierdo, Goiatz Balziskueta, María Sánchez-Bayton Griffith, Nerea Quilez Trasobares, María Ángeles Solinís, Arantxa Isla

**Affiliations:** 1 Department of Pharmacy, Cruces University Hospital, Plaza de Cruces 12, 48903 Barakaldo, Spain; idoia.bilbaomeseguer@osakidetza.eus; 2 Pharmacokinetic, Nanotechnology and Gene Therapy Group (PharmaNanoGene), Faculty of Pharmacy, Centro de Investigación Lascaray Ikergunea, University of the Basque Country UPV/EHU, Paseo de la Universidad 7, 01006 Vitoria-Gasteiz, Spain; ana.alarcia@ehu.eus (A.A.-L.); alicia.rodriguez@ehu.eus (A.R.-G.); 3 Instituto de Investigación Sanitaria Bioaraba, 01009 Vitoria-Gasteiz, Spain; helena.barrasagonzalez@osakidetza.eus (H.B.); FRANCISCOJAVIER.MAYNARMOLINER@osakidetza.eus (J.M.); goiatz.baltziskuetaflorez@osakidetza.eus (G.B.); 4 Intensive Care Unit, Araba University Hospital, Osakidetza Basque Health Service, 01009 Vitoria-Gasteiz, Spain; 5 Inserm U1070: Pharmacologie des Anti-Infectieux, Pôle Biologie Santé, Université de Poitiers, Bâtiment B36, 1 Rue Georges Bonnet, 86022 Poitiers, France; eduardo.asin.prieto@gmail.com; 6 Instituto de Investigación Sanitaria Bioaraba, Microbiology, Infectious Disease, Antimicrobial Agents, and Gene Therapy, 01006 Vitoria-Gasteiz, Spain; 7 Intensive Care Unit, Doce de Octubre Hospital, Avda de Córdoba, s/n, 28041 Madrid, Spain; jasiruci@gmail.com (J.Á.S.-I.); mariabayton@hotmail.com (M.S.-B.G.); nerida.mia@gmail.com (N.Q.T.)

**Keywords:** levetiracetam, augmented renal clearance, intensive care, critically ill patients, population pharmacokinetic, modelling, Monte Carlo simulations, seizure

## Abstract

Levetiracetam is a broad-spectrum antiepileptic drug commonly used in intensive care units (ICUs). The objective of this study is to evaluate the adequacy of levetiracetam dosing in patients with normal or augmented renal clearance (ARC) admitted to the ICU by population modelling and simulation. A multicentre prospective study including twenty-seven critically ill patients with urinary creatinine clearance (CrCl) > 50 mL/min and treated with levetiracetam was developed. Levetiracetam plasma concentrations were best described by a two-compartment model. The parameter estimates and relative standard errors (%) were clearance (CL) 3.5 L/h (9%), central volume of distribution (V1) 20.7 L (18%), intercompartmental clearance 31.9 L/h (22%), and peripheral volume of distribution 33.5 L (13%). Interindividual variability estimates were, for the CL, 32.7% (21%) and, for V1, 56.1% (29%). The CrCl showed significant influence over CL. Simulations showed that the administration of at least 500 mg every 8 h or 1000 mg every 12 h are needed in patients with normal renal function. Higher doses (1500 or 2000 mg, every 8 h) are needed in patients with ARC. Critically ill patients with normal or ARC treated with levetiracetam could be at high risk of being underdosed.

## 1. Introduction

Levetiracetam is a broad-spectrum antiepileptic drug with proven efficacy in treating multiple seizure types, in both the adult and paediatric population. Because of its improved safety profile and ease of use compared to other conventional antiepileptic drugs such as phenytoin, it is frequently used in the treatment of status epilepticus and in seizure prophylaxis after a neurologic injury, being a commonly used treatment in intensive care units (ICUs) [1,2,3].

Levetiracetam has a linear pharmacokinetic profile. It is rapidly and almost completely absorbed when administered orally, with a time to reach the peak concentration (Tmax) of 1–2 h and a high bioavailability (>95%). Its apparent volume of distribution is 0.5–0.7 L/kg with non-significant plasma protein binding (<3%). Renal clearance represents the main elimination mechanism with a 66% of the dose excreted unchanged in urine, which leads to a good correlation between levetiracetam clearance and a patient‘s creatinine clearance (CrCl). Additionally, a fraction of the dose (24%) is eliminated by metabolism through enzymatic hydrolysis of the acetamide group, carried out by a type B esterase, mainly in blood. Clinically relevant interactions are not expected, as this metabolic pathway is only responsible for the metabolism of a small part of the administered dose. Additionally, levetiracetam does not induce or inhibit CYP enzymes resulting in minimal drug-drug interactions. The metabolites have no known pharmacological activity and are renally excreted [1,4,5]. 

There is no clear correlation between levetiracetam serum concentration and efficacy or tolerability. The current reference range for trough concentrations is 12–46 mg/L [6], although some authors have proposed a more modest target range of 6–20 mg/L [7]. The favourable pharmacokinetic profile together with the absence of major drug interactions and broad therapeutic window makes routine therapeutic drug monitoring (TDM) unnecessary. However, TDM, as a way to ensure effective and safe exposures, may be indicated in certain circumstances, such as in patients with altered levetiracetam clearance. This is the case of elderly patients, children, pregnant women, patients with renal insufficiency or critically ill patients [8,9].

In fact, the pharmacokinetic behaviour of levetiracetam has been poorly studied in critically ill patients with augmented renal clearance (ARC). The ARC, defined as a CrCl > 130 mL/min/1.73 m^2^, is present in 20–65% of critically ill patients, being more common in certain conditions, such as traumatic brain injury (TBI) (85%) or subarachnoid haemorrhage (SAH) (100%). Although the physiological mechanism responsible for ARC in critically ill patients is not well-defined, the combination of systemic inflammation coupled with a greater renal functional reserve and together with intensive fluid therapy and the administration of inotropic and vasopressor drugs could explain this phenomenon. The presence of ARC could lead to faster elimination of renally excreted drugs, such as levetiracetam, potentially resulting in subtherapeutic concentrations and poorer clinical outcomes [10,11,12,13]. 

In this regard, the aim of this study is to evaluate the adequacy of levetiracetam dosing for the achievement of therapeutic levels in patients with normal or high renal clearance admitted to the ICU by the characterization of the levetiracetam pharmacokinetics by population modelling and simulation.

## 2. Materials and Methods

### 2.1. Study Design and Patient Population

A multicentric open-label prospective study was conducted in critically ill patients admitted to the ICUs of Araba University Hospital (Vitoria-Gasteiz, Spain) and Doce de Octubre Hospital (Madrid, Spain). Patients were recruited during 2019 and 2020 following a protocol previously approved by the Basque Clinical Research Ethics Committee (EPA2018019 (SP)). The study was carried out in accordance with ICH Guidelines for Good Clinical Practice. Samples and data from patients were provided by the Basque Biobank (www.biobancovasco.org) and were processed following standard operation procedures with appropriate ethical approval. ICU patients were eligible if they were treated with levetiracetam and had a CrCl > 50 mL/min measured in urine. The exclusion criteria were age less than 18 years, pregnancy or hypersensitivity to the active substance or to any of the excipients.

### 2.2. Drug Administration, Sampling Procedure and Analytical Method

Each patient received a dose of 500, 1000 or 1500 mg of levetiracetam every 12 h, as a 30-min intravenous infusion. For each patient, blood samples (3 mL) were taken at 0 h (pre-dose), at the end of the infusion (0.5 h) and at the end of the dosing interval (12 h). Moreover, one sample was taken within the intervals of 1–2 h, 3–5 h and 6–8 h after drug administration. Each sample was immediately centrifuged at 3000 rpm for 10 min to collect the plasma, which was immediately frozen at −20 °C. Within the following week, samples were stored at −80 °C until analysis.

Plasma concentrations of levetiracetam were quantified with a high-performance liquid chromatography (HPLC) assay with ultraviolet detection at a wavelength of 205 nm. The method was validated following the US Food and Drug Administration (FDA) (2018) and the European Medicines Agency (EMA) (2012) guidelines. Separation was performed on a Symmetry^®^ C18 (4.6 mm × 150 mm × 5 µm) column (Waters, Milford, Massachusetts, United States) eluted with ammonium phosphate and acetonitrile (95:5, v:v) mobile phase and it was delivered at 1.2 mL/min. Sample preparation consisted of protein precipitation with acetonitrile and centrifugation for 10 min at 15,000× *g*. The supernatants were then injected into the HPLC system.

The assay was linear over the concentration range from 2 to 100 mg/L. Specificity was assessed using six blank standards and lower limit of quantification (LLOQ) level samples. The chromatograms were checked for interference, with no interference peaks detected at the retention time of levetiracetam. Intra–batch and inter–batch accuracy and precision were evaluated at four different concentration levels (LLOQ and low, middle, and high-quality control) in six replicates. The intra–day and inter–day coefficients of variation (CV) and bias were never above 15%. Stock solution stability, the stability of levetiracetam in storage conditions (at −20 °C for one month and at −80 °C for one year), freeze–thaw stability of the analyte in the matrix from freezer storage conditions to room temperature, and auto-sampler rack stability were also evaluated and confirmed. Levetiracetam substance for standards and quality controls was a reference standard, United States Pharmacopoeia, USP.

### 2.3. Noncompartmental Analysis

PK parameters for levetiracetam were initially explored by noncompartmental analysis using Phoenix 64 (Build 8.3.0.5005, Certara, Princeton, NJ, USA). The following PK parameters were provided for levetiracetam: the area under the concentration-time curve within the dosing interval (AUC_12_), peak plasma concentration (Cmax), apparent systemic clearance (CL), elimination half-life (t_1/2_) and apparent volume of distribution (Vz). Area under the concentration-time curve was calculated using the linear-log trapezoidal rule. Afterwards, the correlation between clearance and CrCl at an individual level was explored. 

Statistical analysis was performed with IBM^®^ SPSS^®^ Statistics for Windows, Version 26. Student t tests were used to compare the pharmacokinetic parameters of levetiracetam between patients in different groups. Statistical significance was assessed at *p* < 0.05.

### 2.4. Pharmacometric Modelling

Nonlinear mixed-effects modelling was implemented in NONMEM (v.7.4), using first-order conditional estimation method with interaction (FOCE+I). On the basis of visual exploration of the data and a review of the literature, one- and two-compartment models were considered to describe the levetiracetam concentration-time data. Regarding the variability model, interindividual variability (IIV) associated with the structural pharmacokinetic parameters was modelled exponentially, whereas the residual variability was tested as either proportional, additive or combined error model. The significance of the off-diagonal elements of the Ω variance–covariance matrix was also explored.

Selection between models was based on the following criteria. First, biological plausibility. Second, a significant reduction in the objective function value (OFV = −2 × log-likelihood). Third, the precision of the parameter estimation expressed as the relative standard error (RSE [%]) and calculated as the ratio between the standard error and the parameter estimate. Fourth, visual inspection of the goodness-of-fit (GOF) plots, including the observed versus individual and population predicted concentration and the residuals plots.

The covariates assessed at baseline evaluated in the analysis included demographic factors (sex, age, height and serum albumin), CrCl (measured in urine), blood chemistry (glucose, albumin, total bilirubin, haemoglobin and leukocytes), acute physiology and chronic health evaluation (APACHE II) and diagnosis. Random effects associated with parameters of interest were plotted versus covariates to explore potential relationships and the Stepwise Covariate Model building tool of Perl speaks NONMEM (v.4.8) was performed as a preliminary selection of covariates. Categorical covariates were modelled as a shift in the typical value for the least common categories, whereas continuous covariates were modelled using linear, exponential or power functions after centring on the median. CrCl was explored as a continuous covariate, but it was also dichotomized into two groups, CrCl < 130mL/min or CrCl ≥ 130 mL/min. Covariates were retained in the model if their inclusion produced a significant decrease of the OFV ≥ 3.84 units (equivalent to *p* < 0.05 for one degree of freedom) in comparison with the previous model without the covariate. This forward inclusion approach was followed by its reverse (backward elimination) removing those covariates, whose elimination did not produce a significant increase of the OFV ≤ 6.63 (equivalent to *p* > 0.01 for one degree of freedom). Therefore, when all the statistically significant covariates were added to the model, each of them was individually removed. If the removal of a covariate was found not to be significant it was dropped in favour of the simpler model.

### 2.5. Final Model Evaluation

GOF plots were used as the first indicator of goodness-of-fit, including the plotting of model-based individual predictions (IPRED) and population predictions (PRED) versus the observed concentrations (DV), conditional weighted residual errors (CWRES) vs time after dose (TAD) and the CWRES vs PRED. The parameter precision was evaluated by running a 2000 sample bootstrap (PsN v.4.8). Finally, a simulation-based model diagnostic to study the performance of the final model, a prediction-corrected Visual Predictive Check (pcVPC), was constructed by replicating 1000 studies with the same design as the original clinical study and representing the 10th, 50th, and 90th percentiles of the observed data and the 95% confidence intervals for the mentioned predicted percentiles, based on the simulated data sets.

### 2.6. Dosing Simulations

Using the same dosing regimens administered to patients, 1000 subjects with different CrCl were simulated (80, 120, 160, 200 and 240 mL/min) to evaluate the impact of the covariate on the levetiracetam clearance. Moreover, stochastic simulations were performed to predict levetiracetam plasma minimum concentrations (Cmin) under various dosing regimens (doses from 500 mg to 2000 mg given at either 12- or 8-h intervals, as a 30-min intravenous infusion) and to estimate the probability of target attainment. The target trough concentrations were 12 to 46 mg/L at steady state as recommended by the International League Against Epilepsy (ILAE). A lower target trough range (>6 mg/L) was also investigated. Simulations with the final model were performed with 1000 virtual subjects with CrCl values within the range from 80 to 240 mL/min. CrCl cut-off values were selected based on the observed distribution of CrCl values of the population included in the study and on the summary of product characteristics of levetiracetam, where dosage adjustments are recommended for CrCl below 80 mL/min, but not above this threshold [1]. Simulations extending infusion time to 2 h were performed in those situations in which target attainment with a minimum probability of 80% was not reached.

## 3. Results

### 3.1. Patient Demographics

**Figure 1:**
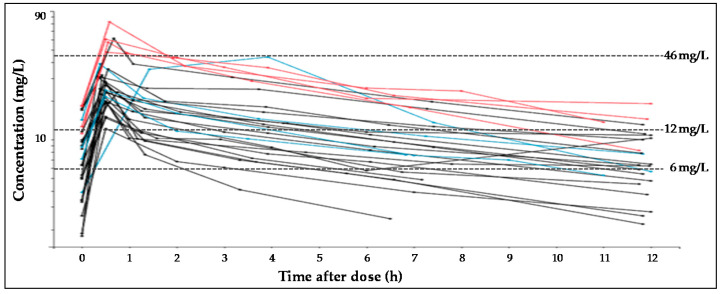
Spaghetti plots for plasma levetiracetam concentration-time profiles, according to dose received by each subject. In black, lines represent profiles after dose of 500 mg, blue lines, 1000 mg and red lines, 1500 mg. Dashed lines represent the target concentration values (6 mg/L, 12 mg/L or 46 mg/L).

Twenty-seven critically ill patients were included in the study. The main diagnoses were haemorrhagic strokes (*n* = 10), trauma (*n* = 8) or other diagnostics such as meningitis, space occupying lesions, convulsive crisis, encephalopathy, arteriovenous malformations or low level of consciousness. Subject characteristics are described in Table 1. A total of 158 plasma samples were analysed, with a median of six, and a minimum of five, plasma samples per patient. Most of the patients (18 out of 27) were treated with 500 mg/12 h of levetiracetam and 10 presented ARC. Levetiracetam was well tolerated, as no evidence of adverse events was recorded, even with the highest dose. Concentration versus time profile of levetiracetam in all the patients is represented in Figure 1. 

### 3.2. Noncompartmental Analysis

**Figure 2:**
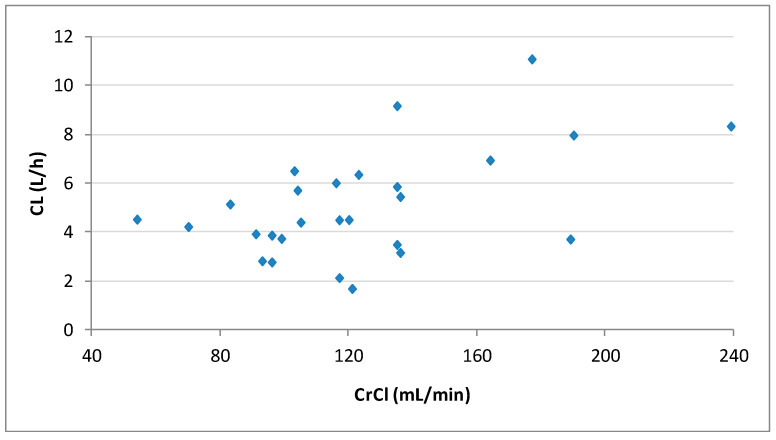
Plot of the individual levetiracetam clearances (CL) calculated by noncompartmental analysis vs. creatinine clearances (CrCl) for the 27 patients.

Pharmacokinetic parameters obtained with noncompartmental analysis are summarized in Table 2. The dose-normalized Cmax and CL were significantly higher in patients with ARC than in those with normal CrCl (*p* > 0.05). Figure 2 shows the correlation between CrCl and levetiracetam clearance calculated by noncompartmental analysis.

### 3.3. Population Pharmacokinetic Modelling

Plasma concentrations were best described by a two-compartment linear model, characterized by drug total body clearance (CL), central volume of distribution (V1), peripheral volume of distribution (V2) and intercompartmental clearance (Q). IIV was exponentially included for CL and V1, and no correlation was detected between the random effects associated with the pharmacokinetic parameters. Residual variability was proportionally modelled. The goodness of fit of the base model was verified by GOF plots.

Both the CrCl, as a continuous variable, and the ARC, as a categorical covariate showed significant influence over CL. CrCl was selected for the final model since the reduction in IIV was greater than with the categorical variable (5.6% vs 3.9%). trauma vs non-trauma diagnosis and APACHE II also showed influence over V1. However, they were eventually excluded from the final model since their individual deletion did not significantly increase the OFV. Therefore, the final model only considered the CrCl as a covariate of the total clearance.

The final model equations were:

$$\mathrm{CL}\left(L/h\right)=\left(3.5+{\left(\frac{\mathrm{CrCl}}{120}\right)}^{2.5}\;\right)\times e^{\eta 1}$$

$$V1\left(L\right)=20.7\times e^{\eta 2}$$

where CL is clearance, CrCl is urinary creatinine clearance, V1 is central volume of distribution, η1 and η2 represent the interindividual variability for CL, and V1, respectively, which followed normal distributions with a mean of 0.

**Figure 3:**
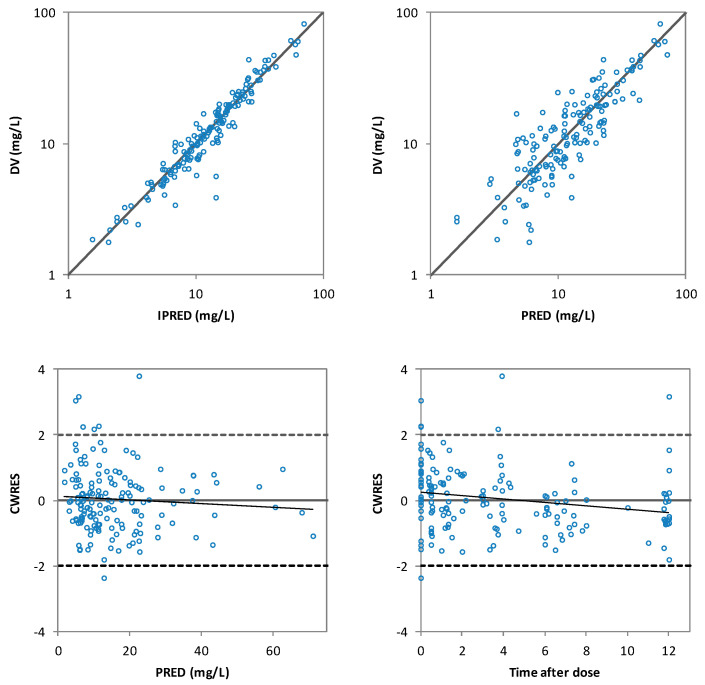
The goodness of fit plots of individual predicted (IPRED) versus the observed (DV) levetiracetam concentrations (**top-left**), population predicted (PRED) versus DV levetiracetam concentrations (**top-right**), conditional weighted residuals (CWRES) versus PRED (**bottom-left**) and CWRES versus time after dose (**bottom-right**) of the final model.

**Figure 4:**
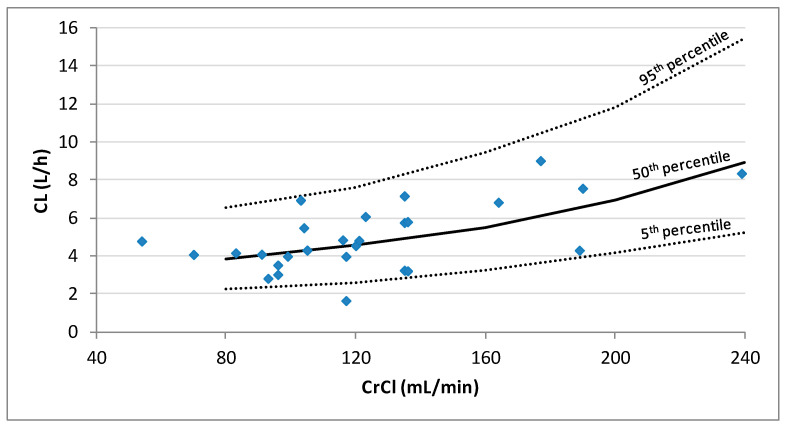
Plot of the individual predicted levetiracetam clearances (CL) estimated by population PK analysis vs. creatinine clearance (CrCl) for the 27 patients. Lines represent the 5th, 50th, and 95th percentiles of 1000 simulations performed at CrCl values of 80, 160, 200, and 240 mL/min.

**Figure 5:**
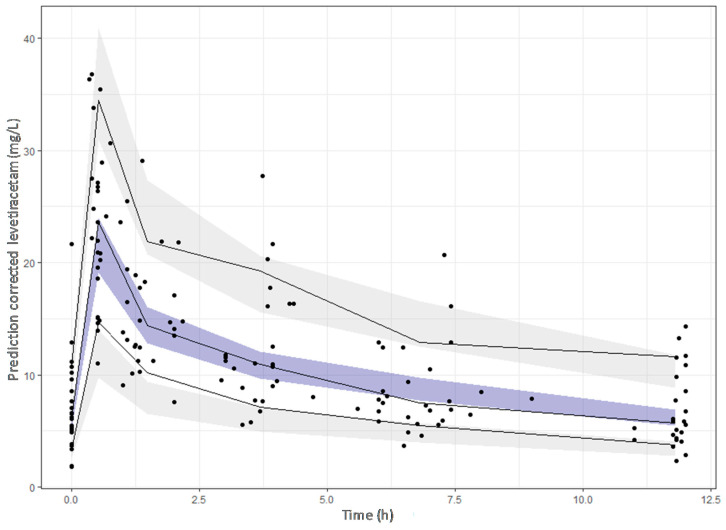
Prediction-corrected visual predictive check of the final model. The dots represent the prediction–corrected concentrations (mg/L). The continuous line represents the 10th, 50th and 90th observed percentiles. Simulation-based 95% confidence intervals for the median and the 10th and 90th percentiles are displayed by dark and light grey shading, respectively.

Inclusion of the CrCl on the CL decreased the unexplained IIV of CL from 38.3% in the base model to 32.7% in the final model and a statistically significant drop of the OFV was obtained with respect to the base model (∆OFV > 6.63). The population PK model and the results of the bootstrap analysis are shown in Table 3. The residual standard errors revealed that all parameters were precisely estimated. Moreover, the estimates of the parameters were very similar to the median values obtained from the bootstrap analysis. Figure 3 displays the GOF plots for the final model. Figure 4 shows the correlation found between CrCl and levetiracetam clearance. The pcVPC, provided in Figure 5, confirmed that the model appropriately predicts both central tendency and variability of the observed concentrations.

### 3.4. Dosing Simulations

Table 4 and Table 5 show the probability of target attainment for simulated patients with different CrCl, calculated as the percentage of virtual subjects (n = 1000) who had levetiracetam trough concentrations above the previously defined values. Considering the target of trough concentrations higher than 12 mg/L, with the twice daily dosing regimen, probabilities higher than 80% were only obtained in patients with no ARC and with the highest doses. More specifically, doses of 1500 mg and 2000 mg every 12 h would be needed for patients with CrCl of 80 and 120 mL/min, respectively. In patients with CrCl of 160 and 200 mL/min, dosing schedules with 8-h interval would be needed (doses of 1500 and 2000 mg, respectively). With those dosing regimens, the probability of Cmin to exceed the value of 46 mg/L is low (<5%) in the respective group of patients. Notably, in patients with CrCl of 240 mL/min the targeted minimum concentration of 12 mg/L was not reached even with doses of 2000 mg every 8 h. Extending the infusion time of the 2000 mg dose to 2 h in this group, did not increase enough the probability of reaching the targeted minimum concentration of 12 mg/L (from 59% to 67%).

When considering the lower target trough concentrations of >6 mg/L twice daily dosing regimens were able to reach the therapeutic interval with a probability greater than 80%, except in patients with CrCl of 240 mL/min, in which dosing every 8 h seemed mandatory. In detail, 1000 mg every 12 h would be suitable for patients with normal renal function, 1500 mg every 12 h for patients with CrCl of 160 mL/min, 2000 mg every 12 h for patients with CrCl of 200 mL/min and 1500 mg every 8 h for patients with CrCl of 240 mL/min.

## 4. Discussion

In this study, a population pharmacokinetic model of levetiracetam in critically ill patients was developed, for a better selection or optimization of the dose regimen, with special focus on ARC condition. ICU patients commonly show altered pharmacokinetics due to their intrinsic heterogeneity and the disease status that can lead to suboptimal drug concentrations. In fact, the high variability observed in levetiracetam concentrations, partially explained by patients’ renal function, suggested the need for dosing optimization in patients with ARC and Monte Carlo simulations revealed the need of high doses to attain the target concentrations.

The ARC condition has recently drawn attention due to its prevalence (present in 20–65% of the patients [10,14] in the intensive care setting), and its potential impact on the elimination of the drugs, especially those primarily eliminated by renal excretion. Pharmacokinetics of renally excreted antimicrobials, such as vancomycin, β-lactams or linezolid, have demonstrated to be significantly modified in patients with ARC [15,16,17,18,19], leading to sub-therapeutic concentrations. In this regard, clinicians should routinely assess the renal function of critically ill patients, by measuring urinary CrCl, not only with the aim of detecting renal impairment, but also, to detect ARC, in order to adjust drug doses. 

Levetiracetam is a widely used drug in ICUs, both in treatment and in prophylaxis of seizures, and is mainly excreted unchanged in urine (66%) making it vulnerable to suffer from increased elimination in patients who display ARC. Nevertheless, the effect of ARC on levetiracetam serum concentrations has been poorly investigated. In a case report, Cook et al. described a 22-year-old girl with severe TBI who displayed ARC. The patient presented a higher than usual systemic clearance of levetiracetam and required significantly higher dose [20]. 

In a study published by Spencer et al. [21], in 12 neurocritical care patients requiring seizure prophylaxis who received 500 mg twice daily, they found a higher levetiracetam clearance and a shorter half-life, compared with previously published results in healthy volunteers. ARC was not present in their population, but there was a statistically significant relationship between the systemic clearance of levetiracetam and estimated CrCl. Just one patient with renal impairment (CrCl 42 mL/min), achieved a steady-state trough concentration greater than 6 mg/L. Recently, two population pharmacokinetic models of levetiracetam in neurocritical patients have been published [22,23]. Sime et al. [22] developed a population pharmacokinetics model in 30 critically ill patients with TBI or SAH without renal disfunction. ARC (urinary CrCl > 130 mL/min/1.73 m^2^) was present in 70% of the patients. Urinary CrCl was found as a covariate that significantly influences levetiracetam clearance, whereas body surface area (BSA) was found to influence levetiracetam clearance, volume of distribution and the absorption rate constant. For every 40 mL/min/1.73 m^2^ increase in urinary CrCl, levetiracetam clearance increased by 50% and the median trough concentrations were reduced by 50%. They performed dosing simulations with dosages ranging from 1000 mg every 12 h to 2000 mg every 8 h and concluded that for urinary CrCl greater than 120 mL/min/1.73 m^2^, none of the simulated regimens had a probability of 80% or above of achieving trough concentrations higher than 12 mg/L. Similarly, Ong et al. [23] have recently developed a population pharmacokinetics model in 20 neurosurgical patients with TBI, SAH or brain tumour resection. ARC (estimated CrCl > 150 mL/min/1.73 m^2^) was present in 30% of the patients. In this study, no covariates were found to significantly influenced levetiracetam pharmacokinetic parameters. They also performed Monte Carlo simulations showing a low probability of reaching trough concentrations > 6 mg/L with the 500 mg twice daily dosing regimen. A dose of 1000 mg twice daily was required to achieve a probability of 80%.

In our study, the pharmacokinetics of levetiracetam were best described by a two-compartment model, agreeing with that reported by Sime et al. [22] and Ong et al. [23]. None of the variables analysed had a significant influence on V1. Trauma diagnosis showed statistical significance at a level of *p* < 0.05, but not at the level of *p* < 0.01, probably because of the scarce number of patients presenting this diagnosis (*n* = 10), and thereby; was not retained in the final model. Other authors have found significant influence of BSA [22,24] or body weight [25] in levetiracetam V1 and/or CL. In a systematic review about levetiracetam pharmacokinetics [25] in paediatric population, healthy subjects or non-critically ill adults, great differences in the volume of distribution, with values from 33 L to 69.9 L (calculated for a 75 kg subject), were reported. In our study, the total volume of distribution was 54.9 L, in the range of most studies, although higher than that observed by Sime et al. (32 L) and Ong et al. (37.2 L) [22,23]. 

In our model, the levetiracetam CL was only dependent on CrCl, which had a great influence on patients with ARC (mean levetiracetam CL increased from 4.5 L/h to 9.2 L/h in patients with CrCl from 120 to 240 mL/min). Sime et al. [22] also included CrCl as a covariate for CL. However, for similar values of CrCl, their model estimates higher levetiracetam clearance. The discrepancies observed between both models could, in part, be due to the differences among the recruited subjects; Sime et al. [22] included only TBI and SAH patients, whereas our population was more heterogeneous according to diagnosis, and also, to age, body weight and CrCl. Ong et al. [23] found similar levetiracetam clearance to that found in our study (3.6 vs. 4.1 L/h for a mean CrCl of 100 mL/min), however, they could not include CrCl as a covariate. This may be, in part, because the subjects included in their study had a narrower range of CrCl than our patients. Moreover, it has to be considered that their patients’ renal function was estimated according to equations, instead of being based on CrCl measured in urine.

Despite the differences in the in the PK parameters, all studies bring out the risk of not achieving the target concentrations in ARC patients. Currently, the most accepted target is to achieve trough concentrations between 12 and 46 mg/L, proposed by ILAE [6], although other authors have proposed lower values. This is the case of the Norwegian Association of Clinical Pharmacology, which recommends target trough concentrations of 5 to 41 mg/L [26]. While ILAE recommendations are based on a retrospective database study that only included the highest doses used by each patient [3], the latter also considered other studies (globally 45% of all samples were below 12 mg/L, and 80% of all samples were between 5 and 25 mg/L) [26]. Moreover, other authors also propose a target trough range of 6–20 mg/L based on typical concentrations values reached with doses ranging from 500 to 1500 mg every 12 h [7].

In our study, a dose of 500 mg every 12 h has shown to be insufficient in critical patients with normal or augmented renal function. In fact, 100% and 67% of these patients had at least one sub-therapeutic level considering the threshold of 12 mg/L or 6 mg/L, respectively. Our results corroborate the need for dose optimization, as the risk for under dosing is highly variable and dependent on the dosing regimen and the renal function of the patients. 

Monte Carlo simulations showed that the maximum dose approved in the summary of product characteristics (1500 mg every 12 h) only guarantees to achieve trough concentration of 12 mg/L in critically ill patients with CrCl ≤ 80 mL/min. In fact, the probability to achieve target trough concentrations higher than 12 mg/L is very low in ARC patients receiving levetiracetam in a twice daily dosing. Doses of 1500 mg and 2000 mg every 8 h are needed to achieve probabilities >80% for individuals with CrCl ≥ 160 and 200 mL/min, respectively, while in patients with CrCl of 240 mL/min, or higher this objective was not reached, even with 2000 mg every 8 h. Several studies have proposed prolonged or continuous infusion to ensure therapeutic concentrations of drugs in patients with ARC [19,27]. We evaluated in patients with CrCl ≥ 240 mL/min if the probability of achieving Cmin target would improve by prolonging the infusion time to 2 h. Monte Carlo simulation showed only a mild improvement. Longer infusions were not studied due to concerns about the stability of levetiracetam solutions at room temperature beyond 4 h [28]. When considering the target trough concentrations of 6 mg/L, probabilities greater than 80% were obtained with 1500 mg every 12 h only for patients with CrCl up to 160 mL/min. Sime et al. [22] reported worse results in their population, as they concluded that even with doses as high as 6 g of levetiracetam per day, trough concentrations within the currently accepted target range were not guaranteed. Therefore, further studies are needed in order to better elucidate the optimal dosing regimen in this population. Moreover, although the role of TDM of levetiracetam has not yet been established, its use in ascertaining compliance and managing patients that are at risk of being over- or under-dosed, such as critically ill patients, would be surely helpful. In addition, it is important to bear in mind that ARC is a dynamic a temporary situation [10], and accordingly, the renal function of the patients should be daily evaluated in order to adjust dosing regimens if needed. 

This study has several limitations. Firstly, this study enrolled a relatively small number of patients, leading to a lack of external validation of the population PK model and limited statistical power. Previous studies were also carried out with a similar number of patients (20–30 patients) [22,23], but a larger sample could allow including any other covariates able to explain some of the remaining variability. In any case, accurate and precise estimates of all parameters were obtained, since a rich sampling strategy was followed in our study. Finally, the lack of consensus about the trough concentration target is a point to address. It would be advisable to determine a well-defined and universally accepted therapeutic range, although it is difficult to establish a correlation between drug concentration and clinical efficacy when levetiracetam is administered prophylactically to prevent seizures. 

## 5. Conclusions

A population pharmacokinetic model has been developed for levetiracetam in critically ill patients with normal or ARC. The pharmacokinetics of the drug were best described by a two-compartment model and CrCl was found to have a significant effect on levetiracetam clearance, which can lead to a high risk of under-exposure, especially in patients with ARC. According to our results, the administration of 500 mg every 12 h could not be enough to achieve the target plasma concentration in the studied population. At least 500 mg every 8 h or 1000 mg every 12 h could be needed in patients with normal renal function. Even the maximum dose approved in the summary of product characteristics (1500 mg every 12 h) could be insufficient in the presence of ARC. However, further studies with a greater number of patients are necessary to determine effective and safety dose regimens in ARC patients.

## Figures and Tables

**Table 1:** Characteristics of the population included in the study.

Covariate	N (%)	Median (Range)
Sex:		
● Male	18 (67)	-
● Female	9 (33)	-
ARC (CrCl > 130 mL/min):		
● Yes	10 (37)	
● No	17 (63)	
Diagnostic:		
● Haemorrhagic strokes	10 (37)	-
● Trauma	8 (30)	-
● Others	9 (33)	-
Age (years)	-	60 (23–81)
Weight (kg)	-	80 (58–115)
Height (cm)	-	168 (148–189)
BSA (m^2^) ^1^	-	1.9 (1.59–2.33)
APACHE II	-	18 (5–35)
CrCl (mL/min) ^2^	-	117 (54–239)
Glucose (mg/dL)	-	142 (91–337)
Albumin (g/dL)	-	3.4 (2.1–3.9)
Total bilirubin (mg/dL)	-	0.6 (0.2–2.1)
Hemoglobin (g/dL)	-	11.6 (6.7–14.5)
Leukocytes (10^9^/L)	-	10.4 (3–24.6)

APACHE: acute physiology and chronic health evaluation; ARC: Augmented renal clearance; BSA: Body Surface Area; CrCl: creatinine clearance. ^1^ Body surface area (Du Bois method) = 0.007184 × Height ^0.725^ × Weight ^0.425^. ^2^ Creatinine clearance= [Urine creatinine (mg/dL) × Volume of urine per minute (mL/min)]/Creatinine plasma level (mg/dL).

**Table 2:** Levetiracetam pharmacokinetic parameters (mean and standard deviation) at steady state following intravenous administration of 500–1500 mg every 12 h to critically ill patients.

	Cmax (mg/L)	Cmax/D (L^−1^)	AUC_12_ (mg·h/L)	AUC_12_/D (h/L)	t_1/2_(h)	CL (L/h)	Vz (L)
No ARC	36.36 (17.93)	0.053 (0.032)	186.49 (97.79)	0.267 (0.118)	8.86 (6.13)	4.28 (1.40)	54.41 (42.79)
ARC	24.25 (12.41)	0.036 (0.011) *	121.05 (66.08)	0.182 (0.081)	7.25 (4.11)	6.51 (2.65) *	61.09 (25.07)

ARC: Augmented renal clearance; Cmax: peak plasma concentration; D: dose; AUC_12_: area under the concentration-time curve within the dosing interval, t_1/2_: elimination half-life; CL: apparent systemic clearance; Vz: apparent volume of distribution; * statistically significant differences between patient with or without ARC (*p* < 0.05).

**Table 3:** Base and final population pharmacokinetic models estimates, shrinkage ^a^ values and bootstrap results.

Parameter	Base Model Estimate (RSE (%))	Final Model Estimate (RSE (%))	Bootstrap Median (95% CI)
CL (L/h) = θnr + (CrCl/120)^θr^	4.6 (8)	-	
θnr	-	3.5 (9)	3.5 (2.8–4.1)
θr		2.5 (17)	2.5 (0.9–3.9)
V1 (L)	20.8 (18)	20.7 (18)	20.8 (13.4–27.7)
Q (L/h)	31.4 (21)	31.9 (22)	30.9 (22.5–47.8)
V2 (L)	34.1 (14)	33.5 (13)	34.2 (19.9–45.4)
IIV_CL (%)	38.3 (19)	32.7 (21)	30.7 (20.2–48.3)
IIV_V1 (%)	54.4 (29)	56.1 (29)	58.0 (22.6–114.0)
RE_proportional (%)	22.3 (15)	22.3 (15)	21.5 (15.7–27.7)

CL, clearance; CrCl, creatinine clearance; V1, central volume of distribution; Q, intercompartmental clearance; V2, peripheral volume of distribution; IIV, inter-individual variability; RE, Residual error; RSE, Relative standard errors; CI, Confidence interval. ^a^ CL ηsh = 2%; V1 ηsh = 23%; εsh = 12%.

**Table 4:** Probability of target attainment based on simulations of the final population model with different doses administered every 12 h. In bold are represented those probabilities ≥80%.

CrCl (mL/min)	Dose (mg)	Perfusion Duration (min)	Daily Dose (mg)	Probability of Cmin (%)		
				>6 mg/L	>12 mg/L	>46 mg/L
**Twice Daily (Tau = 12 h)**						
80	500	30	1000	62	12	0
	1000	30	2000	**93**	60	0
	1500	30	3000	**99**	**85**	3
	2000	30	4000	**100**	**94**	14
120	500	30	1000	43	6	0
	1000	30	2000	**86**	43	0
	1500	30	3000	**95**	72	2
	2000	30	4000	**98**	**85**	6
160	500	30	1000	22	1	0
	1000	30	2000	67	22	0
	1500	30	3000	**87**	51	0
	2000	30	4000	**94**	69	2
200	1000	30	2000	39	6	0
	1500	30	3000	68	25	0
	2000	30	4000	**80**	42	0
240	1500	30	3000	37	7	0
	2000	30	4000	55	15	0

Cmin, Minimum levetiracetam concentration; CrCl, creatinine clearance; Tau, dosing interval.

**Table 5:** Probability of target attainment based on simulations of the final population model with different doses administered every 8 h. In bold are represented those probabilities ≥80%.

CrCl (mL/min)	Dose (mg)	Perfusion Duration (min)	Daily Dose (mg)	Probability of Cmin (%)		
				>6 mg/L	>12 mg/L	>46 mg/L
**Three Times Daily (Tau = 8 h)**						
80	500	30	1500	**94**	51	0
	1000	30	3000	**100**	**93**	5
	1500	30	4500	**100**	**99**	31
120	500	30	1500	**84**	33	0
	1000	30	3000	**99**	**84**	2
	1500	30	4500	**100**	**96**	17
160	500	30	1500	65	12	0
	1000	30	3000	**94**	65	0
	1500	30	4500	**99**	**89**	5
	2000	30	6000	**100**	**97**	17
200	500	30	1500	38	4	0
	1000	30	3000	**83**	39	0
	1500	30	4500	**95**	69	1
	2000	30	6000	**98**	**84**	5
240	1000	30	3000	61	15	0
	1500	30	4500	**80**	38	0
	2000	30	6000	**89**	59	1
	2000	120	6000	**94**	67	1

Cmin, Minimum levetiracetam concentration; CrCl, creatinine clearance; Tau, dosing interval.
